# Employed cancer survivors develop a peer-support intervention to improve social connections: a participatory design study

**DOI:** 10.3389/fragi.2026.1802482

**Published:** 2026-06-26

**Authors:** Alicia G. Dugan, Keith M. Bellizzi, Hannah L. Austin, Sara Namazi, Jennifer M. Cavallari

**Affiliations:** 1 Division of Occupational and Environmental Medicine, University of Connecticut School of Medicine, Farmington, CT, United States; 2 Department of Human Development and Family Sciences, University of Connecticut, Storrs, CT, United States; 3 Department of Psychological Sciences, University of Connecticut, Storrs, CT, United States; 4 College of Health and Wellness, Johnson and Wales University, Providence, RI, United States; 5 Department of Public Health Sciences, University of Connecticut School of Medicine, Farmington, CT, United States

**Keywords:** cancer survivors, community-based participatory research, employment, healthy workplace participatory program, intervention planning, social connection

## Abstract

**Introduction:**

Among the 4.3 million working-age breast cancer survivors in the U.S., employment offers financial stability, purpose, and normalcy. However, many face barriers that hinder workforce participation, including social disconnection. Using a participatory intervention design approach that directly engages survivors, this study aimed to identify root causes of disconnection and prioritize solutions to support psychosocial wellbeing and work retention.

**Methods:**

This study recruited six employed breast cancer survivors for a design team that used the Intervention Design and Analysis Scorecard’s (IDEAS) Steps 1-5 to develop and prioritize intervention options. The team, facilitated by university researchers, met for seven 1-h sessions. Qualitative data from IDEAS worksheets and meeting notes were analyzed.

**Results:**

Survivors identified three causes of social disconnection: (1) changes in self-concept and worldview, (2) symptom-related social withdrawal, and (3) unsupportive responses from others. The team developed and evaluated three intervention models, considering scope, effectiveness, resources, and barriers. An intervention including written materials, a website, a peer-led support group, and a social media forum, was prioritized as the most feasible and impactful option. A follow-up study will pilot-test the intervention to assess effects on wellbeing and work outcomes.

**Discussion:**

The team prioritized a multi-component intervention to foster connection and work engagement primarily targeting individual and interpersonal factors rather than workplace-level factors, likely reflecting the absence of a common employer. Findings highlight social connection as a modifiable target and support further intervention development and pilot-testing, with attention to accessibility and relevance across diverse survivor populations through cultural, economic, and structural adaptations.

## Introduction

Advances in early detection and treatment have resulted in a growing population of working-age breast cancer survivors, estimated at nearly 4.3 million in the United States (Wagle et al., 2025). More than half of breast cancer survivors continue working during treatment, and approximately 80% return to work within 6 months after diagnosis (Chen et al., 2021). Workforce participation is central to financial security, personal identity, and psychosocial wellbeing, yet many survivors encounter persistent social challenges that complicate workforce reentry, retention, and engagement at work (Wu et al., 2024; Zarei et al., 2025).

Social connection is a critical but under-examined determinant of continued employment among cancer survivors. Social support from work colleagues and family members facilitates continued work participation, while personal, organizational, and societal barriers (e.g., fatigue, work overload, lack of social benefits) can disrupt work engagement and increase isolation (Magnavita et al., 2024). These barriers may undermine wellbeing and employment outcomes, particularly as survivors age and manage long-term or late effects of treatment. Consistent with this, evidence shows that social isolation and loneliness are associated with adverse work outcomes, including burnout, low job satisfaction, and reduced productivity (Bryan et al., 2023; Gilmer et al., 2023; McCarthy et al., 2026).

Within the broader social ecology, isolation and loneliness are recognized as widespread life-course concerns in the general population, linked to chronic disease morbidity and premature mortality at levels comparable to major biomedical risk factors (Berkman et al., 2000). They are also associated with poorer physical functioning, greater pain interference, fatigue, anxiety, and depression (Holt-Lunstad et al., 2015; Office of the Surgeon General, 2023; World Health Organization, 2025). Moderate to severe loneliness is commonly reported among cancer survivors, affecting more than one in three (Wheldon et al., 2025). Social relationships influence health through multiple pathways, including behavioral regulation, psychological processes (e.g., stress buffering, emotional wellbeing), physiological systems (e.g., neuroendocrine, immune, and cardiovascular), and access to material and informational resources (Berkman et al., 2000). Reflecting this body of evidence, the American College of Lifestyle Medicine identifies positive social connection as one of six core determinants of health and longevity (alongside healthy nutrition, physical activity, restorative sleep, stress management, and avoidance of risky substances) and underscores its central role in preventing and managing chronic diseases, including cancer, cardiovascular disease, and type 2 diabetes (American College of Lifestyle Medicine, 2026; Bodai et al., 2018).

Existing workplace and survivorship interventions rarely address social disconnection, emphasizing symptom management and individual coping over social and relational processes at work, though it is central to wellbeing (Bakker and Demerouti, 2017; de Boer et al., 2015; Duijtset al., 2017; Karasek and Theorell, 1990; Stergiou-Kita et al., 2014). Moreover, most work-related interventions for survivors are designed by researchers, with limited direct involvement of employed survivors in identifying contextually-relevant barriers and solutions (Bilodeau et al., 2022).

Participatory approaches offer a promising strategy for developing survivor-centered interventions. For example, the Healthy Workplace Participatory Program emphasizes involving members of the workforce who are intended to benefit from an intervention in the intervention design process. A key tool within the program, the *Intervention Design and Analysis Scorecard* (IDEAS), provides a structured process for identifying root causes of worker wellbeing concerns and prioritizing feasible interventions for implementation (Punnett et al., 2020; Robertson et al., 2015). To date, these methods have not been applied to address social disconnection related to workforce engagement among employed breast cancer survivors.

This exploratory study describes a participatory design process in which employed breast cancer survivors identified key sources of social disconnection and prioritized potential interventions to promote psychosocial wellbeing and work engagement. Guided by this approach, the study posed the following questions:

Research Question 1 What are the root causes of social disconnection among employed cancer survivors?

Research Question 2 Which interventions could effectively address the root causes of social disconnection and also have the greatest potential for implementation success, based on their perceived health benefits, reach, needed resources, and feasibility?

This study provides preliminary evidence to inform the development of survivor-informed interventions at the intersection of aging, cancer survivorship, and work.

## Methods

IDEAS is an evidence-based intervention development tool within the Healthy Workplace Participatory Program toolkit developed by the Center for the Promotion of Health in the New England Workplace (CPH-NEW) (Center for the Promotion of Health in the New England Workplace, 2026c; Center for the Promotion of Health in the New England Workplace, 2019). The IDEAS tool is facilitator-guided and engages a design team of workers in identifying root causes of a chosen health-related problem, generating targeted solutions, and evaluating and prioritizing intervention options (Robertson et al., 2015). The IDEAS method ensures interventions are aligned with an integrated health, safety, and well-being approach (Schill and Chosewood, 2013), based on protecting and promoting worker health both on and off the job, through both organizational and individual-level change strategies. This approach is appropriate for the present study because social disconnection among survivors spans both work and non-work contexts.

### The original IDEAS process

In IDEAS, frontline employees serve as members of the design team responsible for developing tailored solutions to workplace health challenges, as subject matter experts of their jobs and work conditions (Center for the Promotion of Health in the New England Workplace, 2026a). IDEAS also specifies involvement of a steering committee of organizational leaders to review and approve proposed design team interventions, and to ensure support and allocation of resources for workplace implementation, particularly in later IDEAS steps (Center for the Promotion of Health in the New England Workplace, 2026b).

A trained facilitator guides the IDEAS process and supports communication between the design team and steering committee, following the Facilitator Manual (Center for the Promotion of Health in the New England Workplace, 2019). The 200-page manual provides extensive guidance, scripts, agendas and worksheets for all seven steps of the IDEAS process. In Step 1, the facilitator guides the design team through a root causes analysis to identify three sub-issues contributing to one major health, safety, or wellbeing concern. In Step 2, the design team establishes a measurable goal and brainstorms solution activities. In Step 3, the design team generates four performance criteria for assessing proposed activities: scope, effectiveness, resources, barriers. In Step 4, the design team evaluates solution activities using the criteria and groups them into three intervention options. In Step 5, intervention options are discussed, rated (high, medium, or low) based on how well they met the criteria, and ranked in order of priority. In Steps 6-7, the steering committee plans, implements, and evaluates interventions.

### Adapted IDEAS process used in this study

Prior research suggests that although the Facilitator Manual is comprehensive and structured, it can be complex, resource-intensive and time-consuming to implement. In real-world workplaces, the IDEAS process requires substantial time and ongoing facilitator support, while design team members face constraints on their engagement such as competing work demands (Cavallari et al., 2021; Sanetti et al., 2022; Strickland et al., 2019). Consistent with these challenges, completing the initial steps (1–5) of the IDEAS process typically takes several months (∼6 months), depending on meeting frequency, organizational support, and the complexity of the health concern being addressed (Cavallari et al., 2021; Namazi et al., 2023). These difficulties have led to calls to better balance the structure of the process with practical feasibility (Cavallari et al., 2021; Sanetti et al., 2022).

For these reasons, IDEAS was adapted for this study. First, the adapted process was limited to IDEAS Steps 1-5, with implementation and evaluation (Steps 6–7) planned for a subsequent study due to the parent project’s time and funding constraints. Additionally, the steering committee was omitted, as participants did not share a common employer. Moreover, because the IDEAS process ended before implementation, the design team assumed responsibility for reviewing and prioritizing interventions, a role typically held by the steering committee in Step 5.

The full set of Facilitator Manual materials was abbreviated to a 20-page Design Team Guide (see Supplementary Datasheet 1). The guide retained core materials for Steps 1-5, including blank worksheets and example worksheets demonstrating the process with a sample problem. Instructions for each step were condensed into one-page summaries, emphasizing the goal of each step and key definitions, rather than providing detailed explanations, agendas, and handouts. Approximately 85% of the content was drawn from the Facilitator Manual, with 15% newly developed. The guide began with the new material, which included an overview of the project’s specific focus on social connection and key findings from a needs assessment survey of employed cancer survivors, as well as an introduction to the IDEAS process. For each step, the guide then provided a single page of concise, tailored instructions focused on social connection among survivors, followed by the original blank worksheets and corresponding example worksheets.

There are additional departures from the original IDEAS process. In the Facilitator Manual, the health problem (e.g., social disconnection) addressed using IDEAS is selected by the design team, but in this study, the researchers selected the problem based on prior survey findings indicating high levels of isolation and loneliness among breast cancer survivors. Additionally, because participants did not share a common workplace, all meetings were conducted virtually. During each session, a trained IDEAS facilitator (university faculty member) guided the process while a university research assistant documented responses in real time on a shared screen. After each meeting, the facilitator and research assistant reviewed audio recordings and transcripts to ensure accuracy, then emailed the completed worksheets to participants for verification before proceeding to the next IDEAS step.

### Recruitment

Employed breast cancer survivors were recruited for a design team to develop interventions to strengthen social connection. Participants who completed the study’s earlier online survey were invited to participate on the design team. The first six respondents were invited to attend seven weekly 1-h meetings, including one introductory session and six sessions dedicated to completing IDEAS Steps 1–5 (some steps require more than one meeting to complete). Participants received $20 for each meeting attended. All study procedures were approved by the University’s Institutional Review Board.

### Data analysis

Data analysis was primarily descriptive and guided by the structured format of the IDEAS tool. Records, including the completed IDEAS worksheets, meeting notes, and transcripts, were reviewed to extract meeting dates, participant attendance, and step-specific outputs, as well as to examine how the intervention developed systematically across steps. Because the IDEAS framework organizes activities into discrete steps, it provided an *a priori* structure for organizing the data. Two researchers reviewed worksheets, extracting key information for each step, including identified wellbeing concerns, root causes, proposed solutions, and decisions about intervention priorities. They then compared summaries and discussed discrepancies through consensus on the finalized data presented in a summary table documenting how the participatory process unfolded over time and culminated in the development of the final “best” intervention.

This approach emphasized summarizing and reporting structured worksheet content rather than applying formal qualitative coding methods, since the data were already generated within a standardized tool. Similar approaches have been used in prior studies of the IDEAS process (Cavallari et al., 2021; Namazi et al., 2023; Strickland et al., 2019). In reporting the narrative results, participant quotes are provided from each IDEAS step to ground findings in survivors’ lived experiences, adding clarity and nuance by showing how they think about and express their concerns in actual language.

Several steps strengthened analytic rigor and transparency. The use of standardized worksheets ensured consistency in how information was collected, and multiple sources were used to provide a comprehensive account of the process. Independent review and consensus between the two researchers reduced bias. All completed worksheets are included in Supplementary Datasheet 2, allowing readers to review how the findings were derived.

## Results

A design team of eight members was convened, which included two academic university researchers: a CPH-NEW faculty member trained as an IDEAS facilitator (AD), and a graduate student research assistant (HA). The six other design team members were employed breast cancer survivors who were female, White, and non-Hispanic. All were married or partnered and reported no childcare responsibilities. Participants ranged in age from 41 to 63 years (M = 55.7, SD = 7.8). Five participants reported having a college or graduate degree, and four reported household incomes above $150,000 (none reported incomes below $75,000).

Half of the participants were diagnosed with cancer early at Stage 1, and half were diagnosed later at Stages 2 or 3. All participants received surgical treatment (five mastectomies, one lumpectomy). Further, participants received one or more additional treatments: four received chemotherapy and endocrine therapy, two underwent reconstruction, and one received external beam radiation. All participants reported working at least 20 h per week at the time that they were diagnosed with cancer. Four of the participants continued to work through cancer treatment, while two stopped working temporarily. At the date of the first design team meeting, two participants were employed full-time, two were employed part-time, one was working on a per diem basis (in an as-needed position), and one had recently retired. Job tenure ranged from 0 to 35 years (M = 18.7, SD = 13.6). Time since ending cancer treatment ranged from 4 months to 5 years (M = 2.2 years, SD = 2.1).

Table 1 summarizes the IDEAS process, including meeting dates, duration, attendance, and outcomes. Participant attendance ranged from 6 to 8 participants attending each meeting (the mean was 7 participants). Seven 1-h meetings took place over 8 weeks, from 30 November 2022 through 18 January 2023.

**TABLE 1:** Design team meeting activities and outcomes for IDEAS Steps 1 through 5.

IDEAS step #	Purpose and outcome of step	Meeting date(s)	# Hours to complete	# Of participants
N/A	**Purpose:** Introductory meeting to meet fellow design team members, understand why social connection is important for employed survivors, and learn about IDEAS tool and design team role	11/30/2022	1	7
Step 1	**Purpose:** Identify a health and safety concern and contributing factors **Outcome:** Concern is social disconnection, isolation, or loneliness among cancer survivors after diagnosis and treatment. Contributing factors (root causes) are: −Reasons related to the permanent transformation of oneself/worldview *(e.g., new priorities, way of relating, state of mental/physical health, identity/appearance)* −Reasons for disconnection originating from survivors’ discomfort *(e.g., poor body image or mental/physical health, fatigue, social distancing/avoidance, comparisons)* −Reasons for disconnection originating from unsupportive people *(e.g., other people’s fear around saying “the wrong thing”, discomfort with cancer, insensitivity, ignorance)*	12/14/2022	1	8
Step 2	**Purpose:** Develop a measurable objective and brainstorm solution activities **Outcome:** Objective is to increase social connections, closeness, and belonging, and to decrease loneliness among survivors after diagnosis and treatment. Solution activities include using different modalities (e.g., via written materials such as checklists and handouts, peer-led survivor support group, social media forum, website, work training) to promote: −Better connection to self *(e.g., guiding self-care and self-improvement related to prioritizing preferred life roles/activities and getting to know/celebrate the “new you”)* −Better connection to inner circle (i.e., friends/family, survivors) *(e.g., pre-worded messages for deep conversations, making plans in advance to connect, participating in activities/events for survivors)* −Better connection to outer circle (i.e., acquaintances, coworkers) *(e.g., a “ready defense” for responding to insensitivity, creating a work plan, choosing a person at work to communicate on your behalf, training for supervisors/HR)*	12/21/2022 12/28/2022	2	8 7
Step 3	**Purpose:** Set selection criteria for selecting and evaluating interventions **Outcome:** Criteria required for a favorable evaluation of intervention includes: *Scope/Impact*. Needs to reach: −Survivors (all cancer types/stages) −Others (A-listers, supervisors, clinicians, acquaintances) Benefits/Effectiveness. Needs to result in: −Increased closeness, belonging, connection to self and others −Decreased loneliness, isolation, disconnection −Decreased stress, anxiety, depression −Decreased job insecurity, absences, turnover intent *Costs/Resources.* Needs resources including: −Key personnel: Intervention/outreach coordinator, SMEs to create written materials, contractors for material formatting, HR staff to deliver training −Funds: Cost of key personnel, written materials, venue/food, shipping, platform fee (i.e., Zoom, if virtual) −Time: kept minimal for both creating interventions (use existing resources where possible) and participating in interventions (written materials, support groups, and workplace training) *Obstacles/Barriers*. Needs to overcome barriers including: −Possibly difficult to reach/engage other cancer types outside of breast cancer, or early stages −Gender norms and lack of time may create obstacles to self-care for women and men in different ways −Feasibility, accessibility, cost −Transportation (if in person), internet/tech issues (if virtual) −Workplace training: Privacy concerns or fear that participation may threaten job	1/4/2023 1/11/2023	1.5	6 7
Step 4	**Purpose:** Apply selection criteria and create 3 intervention options **Outcome:** Three intervention options include: Intervention A: Basic essentials −Written materials only Intervention B: Hybrid −Written materials −Website −Social media forum −Peer-led support group −Intervention C: Comprehensive −Written materials −Website −Social media forum −Professionally-led support group −Workplace training for supervisors	1/11/2023 1/18/2023	1	7 7
Step 5	**Purpose:** Rate and select interventions **Outcome:** List of interventions by priority rank: Intervention B (Hybrid) is first priority −More accessible than intervention a due to communication channels via web/social media and peer-led support group Intervention A (Basic Essentials) is second priority −Cost is low, but may be less effective than intervention B due to limited communication channels Intervention C (Comprehensive) is third priority −Would require significant resources to be successfully implemented, especially staff, money, time	1/18/2023	0.5	7

In Step 1, the team generated a list of three sub-issues with contributing factors (i.e., root causes) that give rise to social disconnection. First, many survivors reported feeling that the cancer experience permanently alters their sense of identity (often accompanied by changes in physical appearance), worldview, and priorities, as well as their physical and mental health needs. These changes can affect how they relate to others, even those with whom they have had close and longstanding relationships.

*“The health effects are permanent. The emotional effects are permanent.”* – Participant 3

*“I’m still here … a reshuffling of my priorities … my family and my loved ones come first. It’s just not as important to me, things like work or how I look. I just want to be alive.”* – Participant 4

Second, survivors’ feelings of self-consciousness about their body image, persistent fatigue, worry, or distress, and unpredictable bouts of physical and emotional discomfort can lead them to cope through social withdrawal or avoidance of social situations.

*“My poor body image … I had a double mastectomy and have not had reconstruction yet. Feeling drained and not looking like myself made me depressed. I just did not want people to see me like that.”* – Participant 2

*“I’m going through something really difficult, and there’s that person over there laughing, and they do not have a care in the world. Maybe, if I’m being honest, it feels like jealousy.”* – Participant 1

Third, survivors reported that people within their social environment including family members, friends, colleagues, and acquaintances can also contribute to disconnection, often unintentionally, through unsupportive responses. This can occur when people show overt or subtle discomfort with cancer as an illness, fear saying the wrong thing, or lack understanding about cancer, resulting in interpersonal awkwardness, insensitivity, or disengagement. These internal and external factors can leave many survivors struggling to maintain meaningful social ties.

*“There's a family member who is very religious and believes that if you ask God to forgive any wrongs that you've done, your cancer will heal naturally, and you will not have to go for any treatments. But I do not have the same belief. This person gave me books to read and everything … and I had to basically tell them, ‘just leave me alone’ because I was getting so frustrated with it all.”* – Participant 4

*“My cancer was being compared with a family member's and a statement was made … ‘Well, you had it so easy by comparison*.*’ It hurt me for somebody else to say ‘Well, yours was not so bad.’”* – Participant 6

In Step 2, the team established a goal of increasing survivors’ sense of connection, closeness, and belonging, while reducing loneliness and social discomfort. The team conceptualized solutions across three interrelated levels: (1) connection to oneself, (2) connection with close social networks, and (3) connection with the broader community. Enhancing connection to oneself involves acknowledging that a cancer diagnosis often precipitates a period of personal and social transformation. This can be facilitated through activities and exercises that nurture self-care, self-awareness, reflection, embodiment, and personal growth.

*“I did massage and Reiki when I was going through my surgeries. It put the focus on something positive for me. I'm feeling horrible and uncomfortable, but this is going to help me and it's going to put me in a position of dealing better.”* – Participant 6

*“There’s a salon and they work with cancer patients going through treatment. If you need to have something done to your hair, if it's falling out and you need to have it cut or anything like that. Or if it's growing back and you need to have it styled. You can get your nails done. It's all these things you can do. It's specifically for people who are still in the treatment process, and it's free.”* – Participant 1

Improving connection to close social ties (the “inner circle”), such as family, friends, and fellow survivors, focuses on proactive communication by anticipating situations and practicing effective responses, and making social plans to engage in meaningful activities and survivor-specific events.

*“I had a friend invite me to a course a culinary course*. *I had a great time and baked I do not know how many dozens of cookies. It was fun being with a friend and meeting new people and learning how to make new cookies from around the world.”* – Participant 4

*“You feel so alone, even though you're completely surrounded by family members and they say they understand… (but) they do not understand the way we (survivors) understand each other.”* – Participant 1

Connection to the broader community and networks (the “outer circle”), which includes acquaintances and coworkers, is supported through activities that ease reintegration into social and occupational environments. These include targeted proactive communication, work planning, readiness to address unsupportive behaviors, designating workplace points of contact, and training workplace supervisors and staff to promote sensitivity and inclusion.

*“I announced (at work) that if I'm quiet and it seems like I'm not engaging, it's not you*. *It's just that I'm not feeling 100%. You do not even have to ask me. My way of handling things is when I do not feel well, I just get very quiet.”* – Participant 4

*“A point person (at work)… if someone's willing to be that person while you're out, to send you a weekly update. Just so when you come back, you have an idea of what's going on.”* – Participant 1

In Step 3, the team developed selection criteria to evaluate potential interventions based on scope, benefits/effectiveness, resource considerations, and potential obstacles. Scope was defined as the intervention reaching survivors across different cancer types and stages, as well as it reaching other key people in the survivor’s life.

*“Any cancer survivor or patient would benefit.”* – Participant 6

*“It's important to catch people early.”* – Participant 1

*“Caregivers are so invested they sometimes need self-care too, so that they're better be able to support (us).”* – Participant 6

*“Children who have parents that are affected with cancer.”* – Participant 1

*“Definitely the workplaces and supervisors.”* – Participant 5

*“Clinicians are experts at how cancer physically affects your body, but it does not hurt to have extra education about how, emotionally and socially, it's affecting us.”* – Participant 1

Effectiveness was defined by an intervention’s potential for more frequent and higher-quality social interactions, greater perceived belonging, and reduced loneliness; improved mental health symptoms (i.e., less stress, anxiety, and depression); and improved work-related outcomes (e.g., perceived job security, fewer missed workdays, and lower turnover intent).

*“Less socially disconnected, more sense of belonging, less loneliness.”* – Participant 7

*“Improve the feeling of job security.”* – Participant 5

*“Anxiety or depression could improve … over the long term.”* – Participant 7

Resource requirements needed for the intervention included funding for staff (i.e., a facilitator to run the group, an expert to develop content), production and dissemination of written and online materials, technology support (i.e., development of a digital platform), and logistical details such as venue. Time was also identified a key resource for both intervention development and participation.

*“People, time, and always money.”* – Participant 6

*“Printing or shipping costs, depending on what you're doing.”* – Participant 5

*“Paying outside contractors, like people to deliver interventions, if you're hiring anybody like a nutritionist or whoever.”* – Participant 6

*“Costs for a virtual platform, if you're using Zoom or something like that.”* – Participant 3

*“Somebody to create a web page or social media.”* – Participant 6

Finally, the team assessed feasibility by identifying potential obstacles; this is intended to enhance planning and mitigate anticipated implementation barriers. Obstacles included limited accessibility for some survivors depending on their cancer type or stage, barriers to self-care based on gender norms or role demands, inaccessibility due to format (i.e., technical difficulties if virtual; transportation difficulties if in-person), and concerns about privacy or workplace stigma.

*“There may be barriers for men. Breast cancer has a lot of public awareness and predominantly affects women, and women are more socially connected and willing to share than men.”* – Participant 6

*“Handouts only have a certain shelf life. They ended up getting crumpled up and thrown away. We deliver materials through social media, YouTube videos, and webinars. (Listening) versus somebody always having to read things. You might also want to consider multilingual delivery.”* – Participant 6

*“When there's technology involved, there's a group of people that just are not able to do it, because they're not technology savvy.”* – Participant 1

*“A private Facebook group.”* – Participant 6

*“You might be afraid of losing your job or sticking your neck out so that they know you're sick, if you do not have that supervisor who has the right balance of care and professionalism.”* – Participant 5

In Step 4, the team developed three tiers of interventions: (A) Basic Essentials, (B) Hybrid, and (C) Comprehensive. (A summary of the three intervention models is shown in Table 1, at Step 4.) Each model represents a progressive increase in scope, complexity, and required resources. The Basic Essentials model consists solely of written materials that offer information and guidance for self-reflection, personal development, and improved coping skills. The Hybrid model adds a website to disseminate written materials and a peer-led support group with social media forum to provide opportunities to discuss topics. The Comprehensive model replaces the peer-led support group with a professionally-facilitated therapy group, and adds a workplace training for supervisors and staff to promote more supportive and inclusive workplaces.

*“We have a very basic version of an intervention. We have a comprehensive version, kind of like a gold standard, optimal ideal intervention with a lot of bells and whistles. And then we have this hybrid somewhere between the two.”* – Participant 7

Written materials and a website were seen as having the broadest reach among survivors across cancer types and stages, as well as being relevant for families, clinicians and employers. The Hybrid model’s support group was seen as particularly beneficial for survivors, while the Comprehensive model was expected to most effectively reach workplace contacts and improve work-related outcomes (e.g., job security, absenteeism, and turnover intentions). All interventions were perceived to enhance connection and to reduce loneliness, stress, anxiety, and depression. Resource demands increased from the Basic Essentials model (lowest) to the Comprehensive model (highest), with the Hybrid model in between. Although both the Basic Essentials and Hybrid models had anticipated barriers, the Comprehensive model was considered to have extensive obstacles and be the most difficult to implement.

In Step 5, the team rated each intervention (high, medium, or low) across scope, effectiveness, resources, and obstacles, and ranked each for implementation priority. The Hybrid model emerged as the preferred option, balancing reach and interpersonal engagement without the extensive resource demands of the Comprehensive model. The Basic Essentials model was cost-effective and easily disseminable but lacked sufficient interpersonal engagement. The Comprehensive model, although potentially most impactful (especially in terms of direct workplace effects), was resource-intensive and less feasible. As a result, the Hybrid model was prioritized as a promising survivor-informed approach that could be expanded as resources and capacity allow.

*“I would go with the hybrid because you have the written materials online and plus you have the group support as well.”* – Participant 4

*“The hybrid is what we could settle on and still get the majority of things done that we wanted to and hopefully develop that final stage later on.”* – Participant 1

## Discussion

This study advances cancer survivorship and employment research by identifying social connection as an important, yet under-addressed, determinant of psychosocial wellbeing and sustained work engagement among employed breast cancer survivors. The findings suggest that social disconnection is multifaceted, arising from three interrelated domains: changes in self-concept, symptom-related withdrawal, and unsupportive responses from others, including in the workplace. Together these key root causes of social disconnection align with prior conceptualizations of loneliness in survivorship as a social-ecological phenomenon (Raque-Bogdan et al., 2019) and represent actionable targets for intervention. The prioritized multi-component intervention was designed to directly address these root causes and was selected based on its perceived health benefits, reach, resource requirements, and feasibility. Although focused on working breast cancer survivors, these findings may have relevance for other populations, including older adults with cancer, for whom social isolation is prevalent.

### Root causes of social disconnection

Survivors described cancer as a disruptive life event that permanently reshaped identity, values, priorities, and relationships with others, including coworkers and supervisors. This aligns with evidence that shifts in work identity and role centrality influence return-to-work processes (Berger et al., 2020). Although identity change is well documented in survivorship research, it is rarely operationalized as a workplace intervention target. Instead, it is often treated as clinical or personal phenomena despite shaping work engagement, belonging, and vocational decisions. Prior research shows that cancer prompts sustained identity work, as individuals renegotiate roles and meaning in response to ongoing health-related uncertainty (Bury, 1982; Charmaz, 1983; Foster et al., 2015). Extending this literature, this study’s findings position identity reconstruction as a work-relevant process influencing social comfort, engagement, and longer-term vocational trajectories. For older employed survivors, these disruptions may intersect with normative age-related transitions such as retirement planning, changing roles, and caregiving, potentially compounding risks for social isolation and a diminished sense of purpose.

Survivors described periods of social withdrawal driven by fatigue, pain, distress, self-consciousness, and unpredictable symptoms. While often framed as maladjustment, participants’ accounts suggest social withdrawal can function as an adaptive coping response that conserves physical and emotional resources and reduces social strain, particularly when symptoms are visible or difficult to explain (Zarei et al., 2025). This aligns with stress and coping frameworks that conceptualize withdrawal as a self-regulating response to conditions of constrained control (Hobfoll, 1989; Lazarus and Folkman, 1984). These findings suggest that workplace strategies should accommodate periods of reduced engagement without pathologizing survivors, while addressing the often-overlooked emotional labor of workplace interactions. Work arrangements such as flextime or remote work may be especially valuable job accommodations. Among older adults, withdrawal may be more consequential, as age-related declines in energy, mobility, and social networks can limit re-engagement and increase the risk of prolonged isolation.

Survivors also identified interpersonal interactions characterized by others’ discomfort, avoidance, insensitivity, or forced optimism as contributing to feelings of disconnection and strain across family and work contexts. This is consistent with prior evidence linking unsupportive interactions (e.g., distancing, minimizing, blaming, awkwardness) with increased psychological distress and physical symptoms, independent of overall stress and supportive interactions (Figueiredo et al., 2004; Ingram et al., 2001). Support may also be mismatched to survivor needs, contributing to loneliness even when survivors have large social networks (Cutrona, 1986; Rini and Dunkel Schetter, 2010). For example, some individuals avoid acknowledging a survivor’s cancer experience because they assume the survivor wishes to preserve a sense of normalcy, whereas the survivor may instead value open recognition of the challenges they have faced (Dugan et al., 2023). For this reason, interventions that facilitate meaningful peer connection and communication may reduce survivors’ loneliness and improve psychosocial outcomes while increasing awareness and use of resources (Poudel et al., 2021; Poudel et al., 2023). For older survivors, these dynamics may be further shaped by ageist assumptions about productivity, adaptability, or retirement readiness, reinforcing exclusion within the workplace and other social contexts.

By identifying unsupportive workplace interactions as a modifiable target, this study expands survivorship and return-to-work interventions beyond accommodation and productivity to include relational and cultural dimensions of work and life, consistent with broader evidence that chronic interpersonal strain can undermine health and wellbeing through multiple mechanisms including physiological, psychological, and health behavioral pathways (Berkman et al., 2000), Together, these results suggest that improving workplace reintegration may be a multifaced endeavor, requiring not only individual-level coping and job modifications, but also organizational-level changes related to communication norms and relational dynamics within the work environment. One successful model is the Dutch return-to-work approach, which emphasizes early work return in some capacity by combining formal supports (a written plan, medical guidance, employer responsibility) with informal supports (supportive managers, flexible coworkers), while giving employees some latitude in how and when they resume work (Beekman, 2024; van Ooijen et al., 2021).

### A promising multilevel solution for social connection

This study’s findings support intervention solutions operating across three nested levels: connection to self, connection with close interpersonal networks, and connection within broader social and workplace environments. Through IDEAS’ structured process and consensus-building, survivors prioritized a Hybrid model for intervention combining educational materials, an informational website, and a peer-led support group with social media forum for ongoing communication with peers between group meetings. The Hybrid model reflected survivors’ preference for an approach that was broad enough to reach diverse survivors while having a relational component to meaningfully reduce disconnection and isolation. The prioritization of the peer-support approach aligns with evidence that online cancer support communities can provide psychosocial benefits such as enhanced social support, emotional wellbeing, and reduced loneliness, particularly for survivors with limited offline support or barriers to in-person participation (Dong et al., 2025; Hong et al., 2012; Westmaas et al., 2020). The choice of the Hybrid model underscores the value of flexible, multi-component interventions that balance reach with social-emotional depth, allowing survivors to engage at different levels of intensity and privacy, when and how they are ready. Such flexibility is critical given evidence that sustained social engagement is a key determinant of healthy aging among older adults living with and beyond cancer.

This study’s findings align with existing research that although single-component interventions such as cognitive behavioral therapy to address maladaptive beliefs, social-emotional learning for skills training, and social prescribing to connect people with groups (Bickerdike et al., 2017; Durlak et al., 2011; Hofmann et al., 2012) can facilitate social connection, they may be most effective when combined, as they address both internal barriers and social opportunities (Hoang et al., 2022; Masi et al., 2011). Although multicomponent approaches are less common, evidence is growing that interventions integrating different modalities for healthy change (e.g., group-based and social identity interventions) are especially powerful (Haslam et al., 2016). These approaches create opportunities for more positive social connections, helping individuals to learn, practice, and build lasting patterns of showing up, deepening relationships, maintaining connections, repairing misunderstandings, and contributing to others (Cruwys et al., 2022; Haslam et al., 2016; Haslam et al., 2019).

### IDEAS adaptation

The study demonstrated successful use of a new Design Team Guide that was adapted from original IDEAS Facilitator Manual content in several ways. The guide retained the core IDEAS process and some key materials (e.g., worksheets, examples) while substantially shortening the process to 20 pages of condensed, one-page step instructions with worksheets. It also provided tailored guidance related to a predetermined health topic of concern within a specific population: social disconnection among employed cancer survivors. This topic was selected by the researchers, rather than by the design team as outlined in the original Facilitator Manual, and the choice was informed by findings from an earlier needs assessment survey of employed survivors. Implementation was conducted fully virtually, with all sessions held online and supported by two academic researchers (trained IDEAS facilitators). The process incorporated real-time shared documentation on IDEAS worksheets during sessions, along with post-session verification procedures, including review of recorded transcripts and participant checks of the worksheets to ensure accuracy.

Adapting IDEAS for survivors working across different employer organizations is a methodological innovation and supports the potential feasibility of intervention design with vulnerable worker populations beyond a single shared workplace. Further, it was responsive to calls for a revised IDEAS process that is less time consuming, easier to understand and use, and more able to engage design team members. In contrast to prior studies that describe a 6-month process (Cavallari et al., 2021; Namazi et al., 2023), this study completed IDEAS Steps 1-5 in a much shorter timeframe (7 hours over 8 weeks), suggesting that the IDEAS process can be adapted to fit more compressed implementation schedules. Participant engagement was strong, with an average attendance of seven of the eight design team members at each meeting.

### Strengths and limitations

Grounded in survivors’ lived experiences, the study’s findings support addressing social connection from an integrated health protection and health promotion perspective, as both an individual and organizational concern (Punnett et al., 2020). Survivors’ emphasis on the “outer circle” (e.g., acquaintances, coworkers) underscores that work-related interventions cannot rely solely on survivor self-management and coping, but must also address how workplace norms, illness-related discomfort, and communication practices shape reintegration and belonging at work.

This study is strengthened by its use of the structured, evidence-based IDEAS participatory framework (Robertson et al., 2015). Although no evaluation of intervention efficacy or feasibility outcomes is presented, the design team’s intervention options were developed and prioritized using IDEAS to deliberately incorporate key determinants of successful implementation, including appropriateness, acceptability, feasibility, perceived effectiveness, broad reach, fit with available resources, and compatibility with user needs (Chaudoir et al., 2013; Dugan and Punnett, 2017). Consistent with diffusion of innovations theory, interventions with these built-in attributes during early design phases strongly influence whether they are later adopted and sustained in real-world settings, often independent of formal efficacy evidence (Rogers, 2003).

However, future research should address the absence of a steering committee. Although the usual employer-based committee was not feasible in this adapted IDEAS process, a small advisory group could be assembled to support intervention development (e.g., content accuracy, platform design) and advise on feasibility, reach, and implementation. This group could also help identify potential barriers, implementation opportunities, and pathways for dissemination across diverse settings. The advisory group could include a mix of relevant partners from both workplace and survivorship contexts such as employer representatives (e.g., human resources or wellness leaders), a cancer advocacy organization (e.g., American Cancer Society), a clinical or survivorship expert (e.g., oncology social worker), and a digital/technology expert.

Several other limitations warrant consideration. Intervention efficacy was not evaluated, and future research should assess intervention impacts on outcomes such as loneliness, belonging, work engagement, absenteeism, retention, and organizational support. The lack of involvement from employers and healthcare providers limits conclusions regarding implementation feasibility in workplace and clinical settings. Although participants were predominantly middle-aged and many were approaching later stages of their careers, likely making the intervention relevant to issues of aging and cancer, the small, relatively homogeneous sample limits generalizability across all survivor populations and occupational contexts. Future research should engage more diverse groups of survivors, and the intervention will likely require cultural, economic, and structural to meet the needs of non-white, lower-income, or caregiving survivors.

### Practical implications

These findings have direct implications for employers, occupational health professionals, and survivorship care providers. From an aging and cancer survivorship perspective, findings underscore social connection as a critical component of healthy aging, with implications not only for work engagement but also for long-term functional independence, mental health, and quality of life among older adults with cancer.

Social connection should be recognized as a modifiable determinant of work engagement, wellbeing, and employment sustainability among cancer survivors. Interventions that combine accessible self-guided resources with structured peer connection may represent a feasible entry point for employers and community health organizations with limited capacity for intensive programming. In addition, workplace initiatives that provide guidance for supervisors and coworkers may reduce unintentional but socially-isolating behaviors and promote inclusive communication norms, consistent with the identification of unsupportive interactions as a modifiable target (Figueiredo et al., 2004; Ingram et al., 2001). Clinically, digital and peer-supported interventions may complement survivorship care by extending psychosocial support beyond active treatment into the prolonged, nonlinear transition back to work. Coordinated implementation may involve collaboration across clinical, vocational, and workplace partners, rather than relying on any single setting or provider.

## Conclusion

This study advances cancer survivorship and employment research by identifying social connection as an important, yet under-addressed, determinant of psychosocial wellbeing and sustained work engagement among employed breast cancer survivors. Findings suggest that social connection is an actionable target for intervention, shaped by factors such as changes in self-concept, symptom-related withdrawal, and unsupportive responses from others. The study also demonstrated the feasibility of an adapted IDEAS process, delivered virtually and with a condensed instructional guide, which enabled completion of Steps 1-5 in 7 hours over 8 weeks. The process had strong participant engagement and yielded a promising multi-component intervention including written materials, a website, a peer-led support group, and a social media forum. Although focused on breast cancer survivors, these findings may extend to other populations, including older adults with cancer, for whom social isolation is prevalent and linked to adverse health and employment outcomes.

## Data Availability

The raw data supporting the conclusions of this article will be made available by the authors, without undue reservation.
