# Supplementary material for: Employed cancer survivors develop a peer-support intervention to improve social connections: a participatory design study
Source: Front Aging. 2026 Jun 26;7:1802482. doi: 10.3389/fragi.2026.1802482 (PMC13350434; doi:10.3389/fragi.2026.1802482)
Supplement: Supplementary file 1 [file DataSheet2.pdf]

Healthy Workplace Participatory Program
CPH-NEW A toolkit for advancing *Total Worker Health*®

# Step 1: Identify Health & Well-being Concern and Root Causes

## General Health and Well-being Concern:

Social Disconnection, Isolation, or Loneliness
Among Cancer Survivors after Diagnosis & Treatment

| Sub-Issue 1: | Sub-Issue 2: | Sub-Issue 3: |
|---|---|---|
| Reasons Related to the Permanent Transformation of Oneself and Worldview | Reasons for Disconnection Originating From Survivors | Reasons for Disconnection Originating From Other People |
| **Contributing Factors:** | **Contributing Factors:** | **Contributing Factors:** |
| • New way of prioritizing time and energy, spent in life roles and activities.<br>• New state of physical and psychological health.<br>• New way of relating to others.<br>• New sense of identity and appearance. | • Survivors feel uncomfortable with physical appearance or body image.<br>• Survivors feel poorly (psychological state).<br>• Survivors feel poorly (physical state).<br>• Survivors feel fatigued and need to conserve energy.<br>• Survivors avoid in-person events due to compromised immune system and avoiding exposure to infectious disease.<br>• Survivors make social comparisons with healthy people that result in upsetting feelings (jealousy, resentment).<br>• Survivors avoid talking about cancer because they do not feel like it or want to protect others from how they really feel. | • People avoid survivors due to fear of cancer or discomfort about not knowing what to say.<br>• People avoid survivors due to discomfort with the ongoing nature of cancer survivorship (they get tired of hearing about it, want things to return to "normal").<br>• People show lack of sensitivity (do not empathize with what survivors are going through or invalidate their feelings).<br>• People show ignorance of cancer treatment and diagnosis (make survivors feel they have brought it on themselves or can control it).<br>• People forget survivors after they have been absent from or outside of work and may exclude them (survivors miss events, get replaced, left behind). |

Healthy Workplace Participatory Program
CPH-NEW A toolkit for advancing *Total Worker Health*

# Step 2: Develop a Measurable Objective and Solution Activities

## Major Health and Well-being Objective:

Increase Social Connections, Closeness, Belongingness & Decrease Loneliness
Among Cancer Survivors after Diagnosis & Treatment

## Solution 1

Better Connection to Self

### Specific Activities For Solution 1:

*Focus on topics related to:*

- **Re-prioritizing life roles.** Invest time/energy into role/activities that improve quality of life.
  - Expand your leisure role. Have more fun. Get adventurous, creative, and playful.
  - Own your self-care role. Treat yourself to things you enjoy. Rest and relaxation.
  - Rethink your work role. Many people want less time at work. Some want more.
- **Transformation of self/worldview.** Get to know, embrace, and celebrate the "new you".
  - Accept your new identity as a survivor
  - Focus on self-discovery
  - Reflect on your life and cancer journey
  - Make meaning out of the trauma
  - Adopt a positive outlook
  - Nurture positive relationships

## Solution 2

Better Connection to 'Inner' Circle
(e.g., friends, family)

### Specific Activities For Solution 2:

*Focus on topics related to:*

- **"Inner Circle" of Friends and Family**
  - Identify/communicate with "A-listers" (people who you are the closest to.)
  - Common things survivors experience and need (including personal boundaries), how they handle things, what they prefer people to do or say.
  - Pre-worded messages of common survivor sentiments that can be hard to articulate
  - Make plans in advance to stay connected (weekly friend dinners, FaceTime calls, etc.)
- **"Inner Circle" of other cancer survivors**
  - Participate in activities or services for cancer survivors (support groups, retreats) or community events or fundraisers supporting breast cancer (team run/walk fundraiser)
  - List of local events where survivors can participate in the above

## Solution 3

Better Connection to 'Outer' Circle
(e.g., acquaintances, coworkers, etc.)

### Specific Activities For Solution 3:

*Focus on topics related to:*

- **Interacting with people who are uncomfortable, insensitive, or uneducated about the cancer experience**
  - What to say when you or other people do not want to talk about cancer, such as "*I'm fine, just tired."*
  - What to say to "label the elephant" when people are uncomfortable, such as *"It's awkward to talk about me having cancer, but I know you're thinking of me and I appreciate it."*
  - What to say for a "ready defense" to set a boundary with insensitive people, such as "*I hear what you are saying, but I need to focus on me and what I'm dealing with*."
- **People at work:**
  - Make a "Work Plan" for when you are absent, so coworkers know what to expect.
  - Workplace training on how to work with or manage staff who are ill (have cancer).

**METHODS TO ADDRESS TOPICS ABOVE:**

- **Create Written Materials:**
  - List of mental health professionals who do therapy with cancer survivors.
  - Instructions for personal journaling, group journaling, or photo-journaling activity centered on specific prompts.
  - Handout of "Tips & Tricks" for self-care (hydration, helpful products, etc.)
  - Instructions for how to create a mission statement/elevator pitch, or use mottos/slogans to keep on positive path
  - Worksheets to guide personal and social development related to re-prioritizing life roles/activities and transformation of self/worldview.

- **Create (Written) Guided Group Activity:**
  - Create format for a "structured support group" for survivors that incorporates all methods (named something like "A New Day", "Reboot" or "Fresh Start").

**METHODS TO ADDRESS TOPICS ABOVE:**

- **Create Written Materials:**
  - Instructions for identifying and communicating with 'A-listers'
  - Checklist with common things survivors experience and need (including personal boundaries), how they may handle things and what they prefer people to do or say.
  - Handout with pre-worded messages that communicate common survivor sentiments that can be hard to articulate
  - Handout (to be updated regularly) with local events where survivors can participate in the above activities/services, community events/fundraisers

- **Create (Written) Guided Group Activity:**
  - Create format for a "structured support group" for survivors that discusses the topics above about connecting with your "Inner Circle" of friends/family and other survivors who have had similar experience

- **Create Website and Social Media Forum** (updated regularly) with local events

  - Choose a point person to handle communication when you are out sick.
  - How to reengage in work activities when treatment is over. *"When I come back, I want to be as engaged going forward as I can be."*
  - "Work version" of what survivors experience, need (including personal boundaries), how they handle things, what they prefer people to do or say.

**METHODS TO ADDRESS TOPICS ABOVE:**

- **Create Written Materials:**
  - List of prepared statements to say when people are uncomfortable, uneducated, or insensitive (possibly on "business cards" to keep in wallet to remind self and share)
  - (Work) Instructions for creating and communicating a "Work Plan" for when you will be out sick.
  - (Work) Instructions for choosing a designated "point person" to coordinate communication when you are out sick.
  - (Work) Instructions for reengaging in work activities when treatment is over.
  - (Work) "Work version" of checklist with common things survivors experience and need (including personal boundaries), how they may handle things and what they prefer people to do or say.

- **Workplace training for managers and other people at work** (mandatory training) **–** on how to work with or manage staff who are ill with specific chronic diseases

Healthy Workplace Participatory Program
CPH-NEW A toolkit for advancing *Total Worker Health*®

# Step 3: Set Criteria for Selecting and Evaluating Interventions

| Scope | Benefits/Effectiveness | Resource Considerations | Obstacles |
|---|---|---|---|
| Who do you want to reach (e.g. one unit or the entire organization)? How many people should be affected? (If you plan a small pilot, describe number in pilot and in long term) | What are the positive outcomes you want to achieve? (both short and long term) | What resources are currently available within the organization that should be considered (e.g. time, money, personnel) ? Are there important parameters or context factors to consider? | What potential barriers exist that may interfere with intervention success?<br><br>**Do not list cost as an obstacle here.** |
| *To be considered successful, the intervention should ideally reach and benefit these cancer survivors…*<br><br>**<u>SURVIVORS:</u>**<br>• **Any Cancer Survivors** – regardless of **cancer type,** and at **all stages** (initial diagnosis, treatment, post-treatment)<br>▪ Catch people early (in early stages of cancer experience)<br><br>**<u>OTHER PEOPLE:</u>**<br>• **Inner Circle A-List/Caregivers:** Supportive people such as family/friends or other survivors<br>• **Workplaces/Supervisors/Coworkers**<br>• **Cancer Centers/(Psychosocial) Clinicians**<br>• **Outer Circle/Acquaintances/Work** | *To be considered effective, the intervention should ideally have these health and well-being benefits…*<br><br>**<u>SHORT TERM:</u>**<br>• **Improve feelings of belonging, closeness, and connection to**:<br>▪ Self<br>▪ Others, community<br>• **Reduce feelings of disconnection**:<br>▪ Lessen loneliness, isolation, and exclusion<br>▪ Remove obstacles to connection<br><br>**<u>MID TO LONG TERM:</u>**<br>• **Decrease mental health symptoms:**<br>▪ Stress (home and work)<br>▪ Anxiety or Depression<br>• **Improve work outcomes:**<br>▪ Lower turnover/absences<br>▪ Perceived job security (i.e., income & insurance security)<br>• **Link with people who provide hands-on assistance/info/resources**<br>▪ Health advocate at insurance company or nurse navigator at hospital | *To be considered acceptable, the intervention should realistically utilize these resources…*<br><br>• **A few key people** *(few as possible):*<br>▪ Person to coordinate intervention delivery / outreach<br>▪ Subject matter experts to create content for written materials<br>▪ Contractors to format materials (marketing, graphic design, web develop/maintain, social media)<br>▪ Professionals (oncology social workers) to deliver expert-led formal support group<br>▪ Survivors to deliver peer-led informal support group<br>▪ HR staff to deliver workplace training for supervisors on managing chronically ill workers<br><br>• **Money** *(as little as possible):*<br>▪ Funds for person to coordinate delivery/ outreach<br>▪ Funds for subject matter experts/contractors<br>▪ In-person format: printing (written materials), venue/food, shipping | *Obstacles/barriers that could interfere with the intervention are…*<br><br>• **Cancer types/stages:** Possibly difficult to reach/engage other cancer types, or early-stage cancer<br>▪ Breast cancer population has a strong network of resources (not so with other cancers)<br><br>• **Sex:** Gender norms and lack of time may create obstacles to self-care for women/men in different ways:<br>▪ Men: Health risk = Masculine; Self-care = Feminine.<br>▪ Women: Self-care = selfish (guilt). Lack of time for self-care because women are more affected by competing demands of work and family<br><br>• **Intervention characteristics:**<br>▪ All interventions need to be accessible, feasible, low cost<br>▪ If virtual: Lack of tech savvy, computer, internet<br>▪ In-person: Lack of transport, need convenient location |

| | | | |
|---|---|---|---|
| | | ▪ Virtual format: platform fee (Zoom)<br><br>• **Time** *(keep things short as possible):*<br>▪ 10 min. to read written materials<br>▪ 6 hrs. for expert/peer support groups (1 hr. per wk. x 6 wks.)<br>▪ ½ day for workplace training<br>▪ Time to create interventions can be shortened if there are existing materials or services available (i.e., for written materials, handouts may already exist from organizations like the American Cancer Society, Cancer & Careers, Livestrong) | ▪ Workplace training: fear of participation threatening job<br>▪ Privacy protection |

Healthy Workplace Participatory Program
CPH-NEW A toolkit for advancing *Total Worker Health*®

# Step 4A: Form Interventions Worksheet

**Major Health and Well-being Objective** (from Step 2):

Increase social connections, closeness, belongingness and decrease loneliness among cancer survivors after diagnosis and treatment

**Key Sub-issues for Intervention** (from Step 2 – list only the sub-issues that are addressed in interventions A, B, or C):

Better connection to self, better connection to 'inner' circle (e.g., friends, family), and better connection to 'outer' circle (e.g., acquaintances, coworkers, etc.)

| Intervention A | Intervention B | Intervention C |
|---|---|---|
| **Title:** Basic Essentials | **Title:** Hybrid | **Title:** Comprehensive |
| **Activities:** | **Activities:** | **Activities:** |
| *Address key sub-issues via:*<br>1. Written Materials | *Address key sub-issues via:*<br>1. Written Materials<br>2. Website & Social Media Forum<br>3. Peer-led Structured Support Group for Survivors | *Address key sub-issues via:*<br>1. Written Materials<br>2. Website & Social Media Forum<br>3. Professionally-led Structured Support Group for Survivors<br>4. Workplace Training For Supervisors |

Healthy Workplace Participatory Program
CPH-NEW A toolkit for advancing Total Worker Health

# Step 4B: Analyze Activities Worksheet

| **Solution Activity** List the activities that you want to include in this intervention: | **Scope** Who will this activity reach? How many people will be affected? | **Benefits/ Effectiveness:** What positive outcomes will be achieved through this activity? (describe both short and long term) | **Resources Needed:** What are the resources needs/costs of this activity? (time, money, personnel) | **Obstacles:** What obstacles or potential barriers could interfere with the success of this activity? |
|---|---|---|---|---|
| **Intervention A** | | | | |
| **Written Materials** | **Survivors** (All cancer types/stages.)<br>**Others** (A-listers, supervisors, clinicians, acquaintances) | Improved connection to self/ others.<br>Decreased loneliness/isolation. | **Resources needed are minimal:**<br>**People** (Coordinating person, subject matter experts)<br>**Money** (for coordinating person, subject matter experts, mailing, printing, postage)<br>**Time** (time to create content, print materials, and disseminate) | **Anticipated obstacles are minimal:**<br>Engages survivors. (All types/stages. Engages women & men.)<br>Fairly accessible, if printed (Is accessible, feasible, low-cost. Possible language barrier.)<br>No privacy concerns |
| **Intervention B** | | | | |
| **Written Materials** | **See above** | | | |
| **Website & Social Media Forum** | **Survivors** (All cancer types/stages.)<br>**Others** (A-listers, supervisors, clinicians, acquaintances) | Improved connection to self/ others.<br>Decreased loneliness/isolation.<br>Decreased stress, anxiety, depression. | **Resources needed are substantial:**<br>**People** (Coordinating person, outreach/marketing person, subject matter experts, contractors)<br>**Money** (for coordinating person outreach/marketing person, subject matter experts, contractors)<br>**Time** (time to create and maintain both website and social media forum) | **Anticipated obstacles are minimal:**<br>Engages survivors. (All types/stages. Engages women & men.)<br>Highly Accessible (Is accessible, feasible, low-cost. Possible language barrier. Participants need to be tech savvy, have computer/internet.)<br>No privacy concerns. |
| **Structured Support Group for Survivors** | **Survivors** (All cancer types/stages.) | Improved connection to self/ others. | **Resources needed are substantial:** | **Anticipated obstacles are limited:**<br>Engages survivors. (All types/stages. Engages women & men.) |

| | | | | |
|---|---|---|---|---|
| | | Decreased loneliness/isolation.<br>Decreased stress, anxiety, depression.<br>Improved job security/turnover. | **People** (Coordinating person, clinician to facilitate (if formal), peer survivor to facilitate (if informal).<br>**Money** (for coordinating person, for clinician to facilitate (if formal), Venue costs (depends on in-person or virtual).<br>**Time** (time to create structure for support group; for survivors to participate) | Less accessible (Is somewhat accessible and feasible. Cost will be higher if expert-led and venue needed. Possible language barrier. If virtual, participants need to be tech savvy, have computer/internet. If in-person, venue needs to be conveniently located, participants need transportation.)<br>Possible privacy concerns. |
| **Intervention C** | | | | |
| **Written Materials** | **See above** | | | |
| **Website & Social Media Forum** | **See above** | | | |
| **Structured Support Group for Survivors** | **See above** | | | |
| **Workplace Training For Supervisors** | **Survivors** (Any cancer type. At all stages.)<br><br>**Others** (Supervisors, Coworkers, Work Acquaintances) | Improved connection to others.<br>Decreased isolation.<br>Decreased stress.<br>Improved job security/turnover. | **Resources needed are extensive:**<br>**People** (Coordinating person, subject matter experts or HR staff to facilitate.)<br>**Money** (for coordinating person, subject matter expert/HR person. venue costs (depends on in-person or virtual).<br>**Time** (time to create content and structure for training session; time for supervisors to participate) | **Anticipated obstacles are extensive:**<br>Does not necessarily engage survivors. (All types/stages. Engages women & men.)<br>Less accessible (Is somewhat inaccessible and unfeasible. Cost will be higher for content development, expert facilitation and work time for training f virtual, participants need to be tech savvy, have computer/internet. If in-person, venue needs to be conveniently located, participants need transportation.)<br>Possible privacy concerns if participation is voluntary. (Make mandatory for all.) |

Healthy Workplace Participatory Program
CPH-NEW A toolkit for advancing *Total Worker Health*

# Step 4C: Apply Criteria for Selecting and Evaluating Interventions

| Instructions to complete this form: | Scope | | Benefits/Effectiveness | | | | Resource Considerations | | | Obstacles | | | Summary | |
|---|---|---|---|---|---|---|---|---|---|---|---|---|---|---|
| | | | Short Term | | Long Term | | | | | | | | | |
| 1) List the selection criteria from Step 3 in the corresponding column.<br>2) List the intervention activities in the appropriate row.<br>3) If an activity meets the selection criteria, place a check-mark in the appropriate cell. If not, leave the cell blank.<br>4) By looking across rows, you can see how well an activity meets all selection criteria.<br>5) By looking down columns, you can assess which activities meet a specific criterion. | Reaches survivors (all cancer types & stages) | Reaches others (A-listers, supervisors, acquaintances) | Improved connection to self and others. | Decreased loneliness and isolation. | Decreased stress, anxiety, depression. | Less turnover intent and perceived job insecurity | Few people (coordinating person, subject matter experts) | Little money (for people, printing, postage) | Less time (to create and implement activities) | May not engage all cancer types & stages, men, non-English speakers | Inaccessible format (if technology based, in-person vs. virtual) | Privacy concerns about participating (if at work, fears about job impact) | **Number of positive criteria (scope, benefits, and resource considerations)** | **Number of negative criteria (obstacles)** |
| **Intervention A** | | | | | | | | | | | | | | |
| Written Materials | ✓ | ✓ | ✓ | ✓ | | | ✓ | ✓ | ✓ | | ✓ | | **7** | **1** |
| **Intervention B** | | | | | | | | | | | | | | |
| Written Materials<br>Website & Social Media Forum<br>Support Group (Peer-led) | ✓ | ✓ | ✓ | ✓ | ✓ | ✓ | ✓ | ✓ | | | | ✓ | **8** | **1** |
| **Intervention C** | | | | | | | | | | | | | | |
| Written Materials<br>Website & Social Media Forum<br>Support Group (Expert-led)<br>Workplace Training | ✓ | ✓ | ✓ | ✓ | ✓ | ✓ | | | | ✓ | ✓ | ✓ | **6** | **3** |

Healthy Workplace Participatory Program
CPH-NEW A toolkit for advancing Total Worker Health

# Step 5: Rate and Select Intervention Options

| Rate the three intervention alternatives as High, Medium, or Low relative to the selection criteria from Step 3. | **Intervention A** **Title: Basic Essentials** (Written materials only) | **Intervention B** **Title: Hybrid** (Written materials + website & social media forum + peer-led support group) | **Intervention C** **Title: Comprehensive** (Written materials + website & social media forum + professionally-led support group + workplace training for supervisors) |
|---|---|---|---|
| **Anticipated Scope (High, Med, Low)→** | **Med** | **High** | **High** |
| **Anticipated Effectiveness/Benefits (High, Med, Low)** | **Med** | **High** | **High** |
| **Anticipated Resources Needed (High, Med, Low)** | **Low** | **Med** | **High** |
| **Anticipated Obstacles (High, Med, Low)** | **Med** | **Med** | **High** |

**Priority rankings of interventions:**

**First: Best option is Intervention B** (Has higher scope and effectiveness than Intervention A due to web/social media presence and peer-led support group; uses fewer resources and has less obstacles than Intervention C.)

**Second:** Intervention A (Requires fewer resources and faces fewer obstacles than Interventions B and C, but has less scope and effectiveness.)

**Third:** Intervention C (Scope and effectiveness are as high as Intervention B, but this is counteracted by high resources and obstacles).

**Additional Notes (optional):**

Low = Fails to, or barely accomplishes selection criteria

Medium = Partly accomplishes selection criteria

High = Meets or exceeds selection criteria
