# Supplementary material for: Employed cancer survivors develop a peer-support intervention to improve social connections: a participatory design study
Source: Front Aging. 2026 Jun 26;7:1802482. doi: 10.3389/fragi.2026.1802482 (PMC13350434; doi:10.3389/fragi.2026.1802482)
Supplement: Supplementary file 2 [file DataSheet1.pdf]

IDEAS Tool
for Enhancing
Social Connections
in Cancer Survivorship:
Design Team Guide

Most of the content in this guide comes from the free, online manual found here:

Center for the Promotion of Health in the New England Workplace (CPH-NEW) (2019). *Healthy Workplace Participatory Program 2.0 Edition Facilitator Manual.* https://www.uml.edu/docs/hwpp_completemanual_final_Remediated_3_23_tcm18-350186.pdf

Introduction: Why is Social Connection a Priority for Employed Survivors? What is the IDEAS Tool and what is a Design Team?

What. Our goal is to better understand the types of support cancer survivors need to continue working or return to work after treatment. We aim to help survivors maintain a good quality of life at work and stay engaged in behaviors that support their health. What we learn will be used to develop workplace and healthcare programs that better support survivors.

Why. Results from our *Cancer Survivors' Work Experiences* survey of workers within five years of a cancer diagnosis show that they have a strong need for support related to their social and emotional well-being, as well as their overall quality of life. Many employed survivors struggle with worry and depression, which are among the biggest sources of stress. Eighty-one percent of survivors said they think about their cancer at least a few times a week and 78% report hiding their true feelings to avoid worrying loved ones. At the same time, relationships often become more meaningful after cancer. Many survivors report positive personal changes, such as becoming more compassionate, less judgmental, and more thoughtful about their relationships. Employed cancer survivors face a range of challenges. Physically, they may experience low energy, increased pain, and trouble sleeping. Emotionally, they may feel stress, anxiety, sadness, or fear, and may spend a lot of time worrying about cancer. Many feel pressured to hide their emotions, which can be exhausting. In terms of coping, some survivors struggle to maintain healthy habits or may feel like giving up, while others take action by asking for help or for workplace accommodations. Financial and insurance concerns can add additional stress.

Key Definitions

IDEAS Tool. The IDEAS Tool (Intervention, Design, and Analysis Scorecard) is a structured, seven-step process developed by the Center for the Promotion of Health in the New England Workplace. The IDEAS tool brings together a "design team" of employees from different levels of an organization to create workplace health interventions. The process focuses on one main health concern at a time and helps the team identify the root causes of health, safety, and well-being issues. By addressing these root causes, the IDEAS process supports the development of targeted, preventive solutions that effectively address the problem.

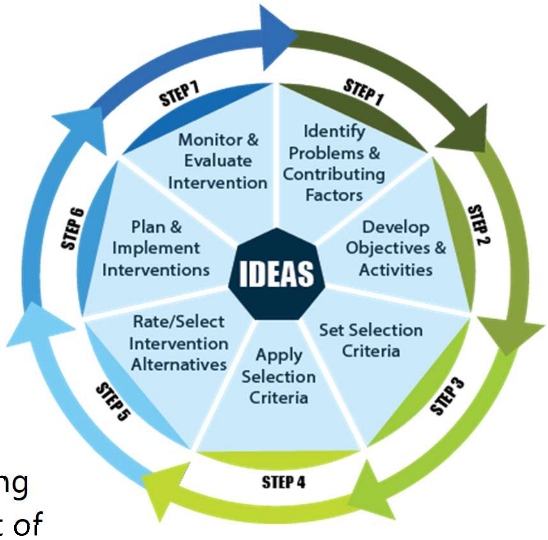

Design Team. A Design Team is a group of workers who share a common understanding of the safety, health, and well-being challenges that employees face. A trained facilitator helps guide the IDEAS process, while team members share their experiences, and work together to develop and propose solutions to improve the work environment and better support employee well-being. In this project, the Design Team is made up of cancer survivors who are managing cancer while remaining employed. As the *Social Connections* Design Team, you will focus on improving psychological and social well-being, as well supporting continued work participation.

Step 1 Instructions: Identify Factors Contributing to Social Disconnection in Cancer Survivors

What. By the end of the meeting, you and your team will have a clear understanding of the agreed-upon contributing factors underlying the primary sub-issues that contribute to poor psychological and social well-being among employed cancer survivors, including at work.

How. Your team will use a root causes analysis technique to brainstorm factors contributing to each of the sub-issues affecting cancer survivors' psychological and social well-being. Your facilitator will assist with the root causes analysis process. In Step 1, your team will discuss the brainstormed items and then select three priority sub-issues that have the strongest effect on cancer survivors' psychological and social well-being. In Step 2, you will generate solutions for the three sub-issues.

Key Definitions for Step 1:

Health, Safety, & Well-being Concern: Anything that places the target population's physical, psychological and/or social well-being at risk.

Sub-Issue: A smaller part or aspect of the health, safety, and well-being concern that helps to make brainstorming of contributing factors easier to think about.

Contributing Factors: Any aspect of a person's life, whether related to themselves, their family life or friendships, work situation, or leisure, that can impact the sub-issue in either a positive or negative way. For example: risky behaviors, interpersonal relationships, work demands, physical environments, availability of resources, etc.

Root Causes Analysis: A process to create a fairly complete list of all of the underlying causes or sources of a health, safety, and well-being concern.

© Center for the Promotion of Health in the New England Workplace, University of Massachusetts Lowell, 2019.

Step 1: Identify Root Causes -- Sub-Issues and Contributing Factors

General Health and Safety Concern:

More employee injuries due to spilled hot coffee over the past six months

**Sub-Issue:**	**Sub-Issue:**	**Sub-Issue:**	**Sub-Issue:**
Coffee cup/lid error	Design of coffee machine	Employee fatigue	Disorganized work space
**Contributing Factors:**	**Contributing Factors:**	**Contributing Factors:**	**Contributing Factors:**
Cups are too thin<br><br>Lid-checks not performed<br><br>Coffee cup sleeves do not easily fit	Coffee machine does not indicate when it is done brewing<br><br>Glass carafes dribble when filling orders	Late-night closers often have opening shift next day<br><br>Average of 4-5 hours of sleep<br><br>Finding shift coverage not standardized<br><br>Understaffed during peak hours	Not enough counter space<br><br>Coffee machine in an inconvenient location<br><br>Coffee machines too close to food prep area

Developed by the Center for the Promotion of Health in the New England Workplace with support from NIOSH grant #U19-OH008857

Healthy Workplace
CPH-NEW Participatory Program

Step 1: Identify Root Causes -- Sub-Issues and Contributing Factors

General Health and Safety Concern:

**Sub-Issue:**	**Sub-Issue:**	**Sub-Issue:**	**Sub-Issue:**
**Contributing Factors:**	**Contributing Factors:**	**Contributing Factors:**	**Contributing Factors:**

Developed by the Center for the Promotion of Health in the New England Workplace with support from NIOSH grant #U19-OH008857

© Center for the Promotion of Health in the New England Workplace, University of Massachusetts Lowell, 2019.

Step 2 Instructions: Develop a Measurable Objective and Brainstorm Solution Activities

What. By the end of the meeting, you and your team will have identified several potential activities for each solution that may offer effective ways to improve psychological and social well-being among employed cancer survivors, including at work.

How. As a team, you will brainstorm solutions (corresponding to the sub-issues identified in Step 1) and create sets of specific activities for achieving each solution.

Key Definitions for Step 2:

Health, Safety, and Well-being Objective: A broad statement of an overall goal for what could be done to improve the health, safety, and well-being concern that was identified in Step 1. The scope of the major health, safety, and well-being goal will be narrowed down into more specific objectives (i.e., "solutions," as defined below), similar to how the health, safety, and well-being concern in Step 1 was narrowed down into "sub-issues".

Solution: A specific, targeted objective for what could be done to accomplish the major health, safety, and well-being objective, making it easier to come up with ideas for specific solution activities.

Activity: A single, specific action that, if implemented, would bring survivors closer to achieving the corresponding solution by addressing contributing factors (as identified in Step 1).

Measurable Improvement: The major health, safety, and well-being objective and its solutions should be "SMART": specific, measurable, achievable, realistic, and time-oriented, to enable us to track progress and know when the goal has been achieved.

© Center for the Promotion of Health in the New England Workplace, University of Massachusetts Lowell, 2019.

Step 2: Develop Measurable Objective and Solution Activities

Major Health and Safety Objective:

Reduce the rate of employee injuries as a result of hot coffee spills

Solution 1:	Solution 2:	Solution 3:	Solution 4:
Improve functionality of coffee cups and lids	Improve safety features of coffee machine	Policy changes/education to reduce employee fatigue	Reorganize workspace to maximize efficiency
**Specific Activities/ Components of Solution 1:**	**Specific Activities/ Components of Solution 2:**	**Specific Activities/ Components of Solution 3:**	**Specific Activities/ Components of Solution 4:**
Order thicker coffee cups/lids with coffee sleeves that fit easily<br><br>Train employees on conducting lid-checks & safe handling of cups	Purchase coffee machines that prevent splashing, dispense directly to cups<br><br>Alternative: Purchase: protective splash guard or better carafes that dribble less	Change scheduling and shift coverage policies<br><br>Provide material on the benefits of sleep/strategies for getting more sleep<br><br>Have an additional employee during peak hours<br><br>Convert walk-in closet to an employee quiet/break room	Store infrequently used supplies to clear counter space<br><br>Hire an ergonomic consultant to reorganize workspace to maximize workflow and usability<br><br>Separate coffee prep and food prep areas

Developed by the Center for the Promotion of Health in the New England Workplace with support from NIOSH grant #U19-OH008857

Step 2: Develop Measurable Objective and Solution Activities

Major Health and Safety Objective:

Solution 1:	Solution 2:	Solution 3:	Solution 4:
**Specific Activities/ Components of Solution 1:**	**Specific Activities/ Components of Solution 2:**	**Specific Activities/ Components of Solution 3:**	**Specific Activities/ Components of Solution 4:**

Developed by the Center for the Promotion of Health in the New England Workplace with support from NIOSH grant #U19-OH008857

© Center for the Promotion of Health in the New England Workplace, University of Massachusetts Lowell, 2019.

Step 3 Instructions: Set Criteria for Selecting and Evaluating Interventions

What. By the end of this step, your team will have developed a set of important factors to consider when evaluating the potential success of each proposed solution activity.

How. As a team, you will discuss the factors that could "make or break" interventions, considering the scope of, expected benefits of, available resources for, and potential barriers facing intervention activities. For all factors, you will decide on the criteria that an activity would have to meet, in order for it to be considered successful or effective. Later in Step 4, you will evaluate whether each activity in the Step 2 Worksheet meets these established criteria.

Key Definitions for Step 3:

Intervention: Generally, an intervention is a planned program, policy, or practice implemented in a work setting to improve worker health, safety or well-being, and often their ability to perform effectively at work. In IDEAS, an intervention is a set of activities that, when implemented together, address the major health, safety, and well-being objective as completely as possible.

Selection Criteria: Standards that the Design Team decides upon to measure the effectiveness or success of their activities and intervention related to four factors: scope, benefits/effectiveness, resources, and obstacles.

Scope: That segment of the population (e.g., individuals, groups) that the intervention is intended to reach and to provide benefits for.

Benefits/Effectiveness: Benefits of any kind that the intervention should provide. For example, better well-being, improved quality of life, improved health or safety, healthier behaviors, better relationships, cost savings, etc. Need to consider both short and long-term effectiveness.

Resources: Estimates by the group of the financial or other types of resources needed and available for an intervention. Lack of current resources should not limit brainstorming of ideas.

Obstacles: Anything that is likely to be a barrier to or work against the interventions being considered; for example, inability to communicate the intervention to the intended population, difficulty in scheduling meetings, people's resistance to change, etc.

Step 3: Set Criteria for Selecting and Evaluating Interventions

Scope	Benefits/Effectiveness	Resource Considerations	Obstacles
Who do you want to reach (e.g. one unit or the entire organization)? How many people should be affected? (If you plan a small pilot, describe # in pilot and in long term)	What are the positive outcomes you want to achieve? (both short and long term)<br><br>**SHORT TERM**	What resources are currently available within the organization that should be considered? (e.g. time, money, personnel). Are there important parameters or context factors to consider?	What potential barriers exist that may interfere with intervention success?<br><br>**Do not list cost as an obstacle here.**
All coffee servers at this franchise location should benefit from changes being made.<br><br>It's desirable to make changes that could be adopted by other franchise locations too	Increased employee satisfaction and less stress<br><br>Better employee comfort<br><br>Higher customer satisfaction<br><br>**LONG TERM**<br><br>Fewer burn injury reports<br><br>Fewer reports of employee fatigue on annual employee survey	Employees semi-annual training can be used to educate on spills<br><br>Costs for supplies not to exceed 1% over existing budget for sustainability<br><br>Any remodeling or new equipment installation should not stop service<br><br>Require a one-time change	Eco-conscious customers want recyclable cups/lids<br><br>Employees may be resistant to changes in workspace design<br><br>Implementing scheduling procedure changes may be difficult<br><br>Fear of more workers compensation claims (due to increased awareness)
	**Short term examples**: Increased knowledge, behavior change, participation, satisfaction<br>**Long term examples**: Improved health, lower claims/costs, more productive	Design Teams may propose interventions that exceed the resources currently available if the benefits justify the costs. Resources should not limit brainstorming.	

Developed by the Center for the Promotion of Health in the New England Workplace with support from NIOSH grant #U19-OH008857

© Center for the Promotion of Health in the New England Workplace, University of Massachusetts Lowell, 2019.

Healthy Workplace
CPH-NEW Participatory Program

Step 3: Set Criteria for Selecting and Evaluating Interventions

Scope	Benefits/Effectiveness	Resource Considerations	Obstacles
Who do you want to reach (e.g. one unit or the entire organization)? How many people should be affected? (If you plan a small pilot, describe # in pilot and in long term)	What are the positive outcomes you want to achieve? (both short and long term)	What resources are currently available within the organization that should be considered? (e.g. time, money, personnel). Are there important parameters or context factors to consider?	What potential barriers exist that may interfere with intervention success?<br><br>**Do not list cost as an obstacle here.**
	**SHORT TERM**		
	**LONG TERM**		
	**Short term examples**: Increased knowledge, behavior change, participation, satisfaction<br>**Long term examples**: Improved health, lower claims/costs, more productive	Design Teams may propose interventions that exceed the resources currently available if the benefits justify the costs. Resources should not limit brainstorming.	

Developed by the Center for the Promotion of Health in the New England Workplace with support from NIOSH grant #U19-OH008857

© Center for the Promotion of Health in the New England Workplace, University of Massachusetts Lowell, 2019.

Step 4 Instructions: Apply Selection Criteria to Solution Activities and Create Intervention Options

What. By the end of this step, your team will have evaluated all the activities created in the Step 2 Worksheet using the selection criteria from the Step 3 Worksheet. These activities will be grouped into three intervention options that will be rated and prioritized in Step 5 as interventions for implementation in a follow-up study.

How. Your team will evaluate all activities listed in the Step 2 Worksheet and discuss how well each activity meets the Step 3 criteria. IDEAS Step 4 is the most complex step and requires at least two meetings. In this step, you will follow a structured process using three worksheets: (1) group solution activities together into three intervention options (*Worksheet 4A*), (2) analyze each intervention using the Step 3 criteria (*Worksheet 4B*), and (3) apply the criteria to each intervention option (*Worksheet 4C*).

Key Definitions for Step 4:

Activity: A single, specific action that, if implemented, would bring survivors closer to achieving the corresponding solution by addressing contributing factors.

Intervention: Generally, an intervention is a planned program, policy, or practice implemented in a work setting to improve worker health, safety or well-being, and often their ability to perform effectively at work. In IDEAS, an intervention is a set of activities that, when implemented together, address the major health, safety, and well-being objective as completely as possible.

Selection Criteria: Standards that the Design Team decides upon to measure the effectiveness or success of their activities and intervention related to four factors: scope, benefits/effectiveness, resources, and obstacles.

Scope: That segment of the population (e.g., individuals, groups) that the intervention is intended to reach and to provide benefits for.

Benefits/Effectiveness: Benefits of any kind that the intervention should provide. For example, better well-being, improved quality of life, improved health or safety, healthier behaviors, better relationships, cost savings, etc. Need to consider both short and long-term effectiveness.

Resources: Estimates by the group of the financial or other types of resources needed and available for an intervention. Lack of current resources should not limit brainstorming of ideas.

Obstacles: Anything that is likely to be a barrier to or work against the interventions being considered; for example, inability to communicate the intervention to the intended population, difficulty in scheduling meetings, people's resistance to change, etc.

© Center for the Promotion of Health in the New England Workplace, University of Massachusetts Lowell, 2019.

SAMPLE COMPLETED WORKSHEET

Step 4A: Form Interventions Worksheet

Major Health, Safety, & Well-Being Objective (from Step 2)

Reduce the rate of employee injuries as a result of hot coffee spills

Key sub-issues for intervention (from Step 2 – list only the sub-issues that are addressed in interventions A, B, or C)

Coffee cup/lid error; Design of coffee machine; Policy changes/education to reduce employee fatigue; Disorganized work space

Intervention A	Intervention B	Intervention C
**Title:**	**Title:**	**Title:**
Improve functionality of coffee cups and lids	Improve safety features of coffee machine	Reorganize workspace to maximize efficiency
**Activities**	**Activities**	**Activities**
1. Order thicker coffee cups/lids with coffee sleeves that fit easily	1. Purchase coffee machines that prevent splashing, dispense directly to cups	1. Store infrequently used supplies to clear counter space
2. Train employees on conducting lid-checks & safe handling of cups	2. Purchase protective splash guard or better carafes that dribble less	2. Hire an ergonomic consultant to reorganize workspace to maximize workflow and usability
3.	3.	3. Separate coffee prep and food prep areas
4.	4.	4.
5.	5.	5.

Developed by the Center for the Promotion of Health in the New England Workplace with support from NIOSH grant #U19-OH008857

159 © Center for the Promotion of Health in the New England Workplace, University of Massachusetts Lowell, 2019.

Step 4A: Form Interventions Worksheet

Major Health, Safety, & Well-Being Objective (from Step 2)

Key sub-issues for intervention (from Step 2 – list only the sub-issues that are addressed in interventions A, B, or C)

Intervention A	Intervention B	Intervention C
**Title:**	**Title:**	**Title:**
**Activities**	**Activities**	**Activities**
1.	1.	1.
2.	2.	2.
3.	3.	3.
4.	4.	4.
5.	5.	5.

Developed by the Center for the Promotion of Health in the New England Workplace with support from NIOSH grant #U19-OH008857

SAMPLE COMPLETED WORKSHEET

Step 4B: Analyze Activities Worksheet

**Solution:**	Reorganize workspace to maximize efficiency			
**Solution Activities**<br>List the activities that you want to include in this intervention	**Scope**<br>Who will this activity reach? How many people will be affected?	**Benefits/ Effectiveness**<br>What positive outcomes will be achieved through this activity? (short and long term)	**Resources Needed**<br>What are the resources needs/costs of this activity? (time, money, personnel)	**Obstacles**<br>What obstacles or potential barriers could interfere with the success of this activity?
Store infrequently used supplies to clear counterspace	All employees	- More counter space decreases clutter and spilling	--2-4 hrs employee planning time<br>-$100 for storage shelves	-May disadvantage shorter employees
Hire an ergonomic consultant to reorganize workspace and workflow	All employees	-Fewer collisions between workers<br>-Fewer spills<br>-Faster, easier order filling	-$250/ 1.5 hour consulting fee<br>-Potential cost (?) of new equipment based on assessment	-Managers may worry that consultant will recommend expensive changes
Separate food prep from coffee prep area	All employees	-Fewer claims for burns	-- $3,000 for remodeling new food prep area	-Time and hassle of hiring and supervising a contractor<br>-Impact to orders during construction

Developed by the Center for the Promotion of Health in the New England Workplace with support from NIOSH grant #U19-OH008857

© Center for the Promotion of Health in the New England Workplace, University of Massachusetts Lowell, 2019.

© Center for the Promotion of Health in the New England Workplace, University of Massachusetts Lowell, 2019.

Step 4B: Analyze Activities Worksheet

		**Solution:**		
**Solution Activities**<br>List the activities that you want to include in this intervention	**Scope**<br>Who will this activity reach? How many people will be affected?	**Benefits/ Effectiveness**<br>What positive outcomes will be achieved through this activity? (short and long term)	**Resources Needed**<br>What are the resources needs/costs of this activity? (time, money, personnel)	**Obstacles**<br>What obstacles or potential barriers could interfere with the success of this activity?

Developed by the Center for the Promotion of Health in the New England Workplace with support from NIOSH grant #U19-OH008857

SAMPLE COMPLETED WORKSHEET

Step 4C: Apply Criteria for Selecting & Evaluating Interventions

**Instructions to complete this form:** 1) List the selection criteria from Step 3 in the corresponding column. 2) List the intervention activities in the appropriate row. 3) If an activity meets the selection criteria, place a check-mark in the appropriate cell. If not, leave the cell blank. 4) By looking across rows, you can see how well an activity meets all selection criteria. 5) By looking down columns, you can assess which activities meet a specific criterion.	**Scope**		**Benefits / Effectiveness**				**Resource Considerations**				**Obstacles**				**Summary**	
			**Short Term**		**Long Term**											
	All coffee servers at this franchise location should benefit from changes being made.	It's desirable to make changes that could be adopted by other franchise locations too.	Increased employee satisfaction and less stress	Better employee comfort	Fewer burn injury reports	Fewer reports of employee fatigue on annual employee survey	Employees semi-annual training can be used to educate on spills	Costs for supplies not to exceed 1% over existing budget for sustainability	Any remodeling or new equipment installation should not stop service	Require a one-time change	Eco-conscious customers want recyclable cups/lids	Employees may be resistant to changes in workspace design	Implementing scheduling procedure changes may be difficult	Fear of more workers compensation claims (due to increased awareness)	**Number of positive selection criteria (scope, benefits/effectiveness, and resource considerations)**	**Number of negative selection criteria (obstacles)**
**Intervention A Activities**																
-Order thicker coffee cups/lids with coffee sleeves that fit easily<br>-Train employees on conducting lid-checks & safe handling of cups	√	√	√		√			√	√	√	√				**7**	**1**
**Intervention B Activities**																
-Purchase a coffee machines that prevent splashing, dispense directly to cups<br>-Alternative: Purchase protective splash guard or better carafes that dribble less	√				√				√	√		√		√	**4**	**2**
**Intervention C Activities**																
-Store infrequently used supplies to clear counter space<br>-Hire an ergonomic consultant to reorganize workspace to maximize workflow and usability<br>-Separate coffee prep and food prep areas	√					√			√	√			√		**4**	**1**

Developed by the Center for the Promotion of Health in the New England Workplace with support from NIOSH grant #U19-OH008857

© Center for the Promotion of Health in the New England Workplace, University of Massachusetts Lowell, 2019.

Step 4C: Apply Criteria for Selecting & Evaluating Interventions

Instructions to complete this form:	Scope		Benefits / Effectiveness				Resource Considerations				Obstacles				Summary	
			Short Term		Long Term											
1) List the selection criteria from Step 3 in the corresponding column.<br>2) List the intervention activities in the appropriate row.<br>3) If an activity meets the selection criteria, place a check-mark in the appropriate cell. If not, leave the cell blank.<br>4) By looking across rows, you can see how well an activity meets all selection criteria.<br>5) By looking down columns, you can assess which activities meet a specific criterion.															Number of positive selection criteria (scope, benefits/effectiveness, and resource considerations)	Number of negative selection criteria (obstacles)
**Intervention A Activities**																
**Intervention B Activities**																
**Intervention C Activities**																

Developed by the Center for the Promotion of Health in the New England Workplace with support from NIOSH grant #U19-OH008857

© Center for the Promotion of Health in the New England Workplace, University of Massachusetts Lowell, 2019.

Step 5: Rate and Select Interventions

What. By the end of this step, your team will have rated the set of proposed interventions designed to benefit the psychological and social well-being of employed cancer survivors, including at work. Priority intervention options will be implemented in a follow-up study.

How. For each selection criteria, compare the important details across all intervention options, and then rate them as High (H), Medium (M), or Low (L). Next come up with a priority ranking (i.e., 1st, 2nd, 3rd), in terms of which intervention should be implemented first. Keep in mind:

- Some selection criteria may be more important than others (e.g., *scope* may be more important than *cost* for a small intervention), so a simple average of all of the ratings is usually not very useful.
- Instead, consider placing more weight on those ratings that matter most, and prioritize intervention options accordingly.
- The group should reach a consensus about the final rank order.

Key Definitions for Step 5:

A HIGH rating (H): An "H" is used to indicate that the activities that make up an intervention meet or exceed what is stated in the selection criteria.

A MEDIUM rating (M): An "M" is used to indicate that the activities that make up an intervention only partly accomplish what is stated in the selection criteria.

A LOW rating (L): An "**L**" is used to indicate that the activities that make up an intervention fail to accomplish, or barely accomplish what is stated in the selection criteria.

Priority Ranking: Intervention options should be ranked after deciding how much weight to place on each of the selection criteria and how well each intervention option addresses the health, safety, and well-being concern.

179

© Center for the Promotion of Health in the New England Workplace, University of Massachusetts Lowell, 2019.

Step 5: Rate and Select Intervention Options

SAMPLE COMPLETED WORKSHEET	Intervention A<br>Title:	Intervention B<br>Title:	Intervention C<br>Title:
Rate the three intervention alternatives as High (H), Medium (M), or Low (L) relative to the selection criteria from Step 3.	Improve functionality of coffee cups and lids	Improve safety features of coffee machine	Reorganize workspace to maximize efficiency
**Anticipated Scope (L/M/H)**	L	H	H
**Anticipated Benefits (L/M/H)**	L/M	H	M
**Anticipated Resources Needed (L/M/H)**	L	M/H	M
**Anticipated Obstacles (L/M/H)**	H	H	L

Topics to discuss with Design Team regarding proposed intervention (optional):

Intervention A: SC agrees with ratings for anticipated benefits and resources. However, using thicker coffee cups materials may conflict with eco-friendly brand. We rated "high" for obstacles because we might lose customers. SC would like to research compostable cup options if DT feels this option is essential.
Intervention B: SC agrees this intervention may have the greatest impact. However, we are concerned about the time and effort to find an alternative coffee machine design and if it will impact coffee flavor. Are DT members willing to gather more info so SC can make an informed decision before purchasing new machines?
Intervention C: This seems like a win-win. All employees benefit and the obstacles are quite low.

Intervention(s) selected for implementation:

We recommend proceeding with option C first, then possibly implementing other options based on the results of additional research.

Developed by the Center for the Promotion of Health in the New England Workplace with support from NIOSH grant #U19-OH008857

178 © Center for the Promotion of Health in the New England Workplace, University of Massachusetts Lowell, 2019.

Step 5: Rate and Select Intervention Options

	Intervention A<br>Title:	Intervention B<br>Title:	Intervention C<br>Title:
Rate the three intervention alternatives as High (H), Medium (M), or Low (L) relative to the selection criteria from Step 3.			
**Anticipated Scope (L/M/H)**			
**Anticipated Benefits (L/M/H)**			
**Anticipated Resources Needed (L/M/H)**			
**Anticipated Obstacles (L/M/H)**			

Topics to discuss with Design Team regarding proposed intervention (optional):

Intervention(s) selected for implementation:

Developed by the Center for the Promotion of Health in the New England Workplace with support from NIOSH grant #U19-OH008857
